# Addition of chemoradiotherapy to palliative chemotherapy in de novo metastatic nasopharyngeal carcinoma: a real-world study

**DOI:** 10.1186/s12935-022-02464-7

**Published:** 2022-01-24

**Authors:** Shuo-Han Zheng, Yu-Tong Wang, Song-Ran Liu, Zi-Lu Huang, Guan-Nan Wang, Jin-Tao Lin, Shi-Rong Ding, Chen Chen, Yun-Fei Xia

**Affiliations:** 1 State Key Laboratory of Oncology in South China, Collaborative Innovation Center for Cancer Medicine, Guangdong Key Laboratory of Nasopharyngeal Carcinoma Diagnosis and Therapy, Sun Yat-Sen University Cancer Center, Guangzhou, China; 2 Department of Radiation Oncology, Sun Yat-Sen University Cancer Center, Guangzhou, China; 3 Zhongshan Medical College, Sun Yat-Sen University, Guangzhou, China; 4 Department of Pathology, Sun Yat-Sen University Cancer Center, Guangzhou, China; 5 Department of Head & Neck Surgery, Sun Yat-Sen University Cancer Center, Guangzhou, China

**Keywords:** Nasopharyngeal carcinoma, Metastasis, Chemotherapy, Radiotherapy

## Abstract

**Background:**

To determine whether concurrent chemotherapy is necessary during locoregional radiotherapy (RT) after palliative chemotherapy (PCT) in patients with de novo metastatic nasopharyngeal carcinoma (mNPC).

**Methods:**

A total of 746 patients with mNPC from 2000 to 2017 at our hospital were retrospectively reviewed. Among them, 355 patients received PCT followed by RT. Overall survival (OS) and progression-free survival (PFS), including locoregional progression-free survival (LRPFS) and distant progression-free survival (DPFS) were estimated with the Kaplan–Meier method and log-rank test. Cox proportional-hazards models, landmark analyses, propensity score matching, and subgroup analyses were used to address confounding.

**Results:**

Of the patients included in our study, 192 received radiotherapy alone after PCT (PCT + RT), and 163 received concurrent chemoradiotherapy after PCT (PCT + CCRT). The prognosis of PCT + CCRT was significantly better than that of PCT + RT (5 year OS, 53.0 vs 36.2%; P = 0.004). After matching, the 5 year OS rates of the two groups were 55.7 and 39.0%, respectively (P = 0.034) and the median DPFS were 29.4 and 18.7 months, respectively (P = 0.052). Multivariate Cox regression analysis indicated that PCT + CCRT was an independent favorable prognostic factor (P = 0.009). In addition, conducting concurrent chemoradiotherapy after 4–6 cycles of PCT or conducting concurrent chemotherapy with single-agent platinum was associated with significant survival benefit in the matched cohort (5 year OS rate, 60.4 or 57.4%, respectively). The survival difference between groups remained significant when evaluating patients who survived for ≥ 1 year (P = 0.028).

**Conclusions:**

The optimal treatment strategy of mNPC is the combination of PCT followed by concurrent chemoradiotherapy. More specifically, concurrent chemoradiotherapy with single-agent platinum after 4–6 cycles of PCT is suggested.

**Supplementary Information:**

The online version contains supplementary material available at 10.1186/s12935-022-02464-7.

## Background

Nasopharyngeal carcinoma (NPC) is a malignant tumor of the nasopharyngeal epithelium that exhibits an unbalanced geographical distribution [1]. In endemic regions, especially in South China, the worldwide age-standardized incidence rate of NPC is up to 25.39/100 000 person-years [2]. Among them, 4–10% of patients present with de novo metastatic nasopharyngeal carcinoma (mNPC), and the 5-year survival rate of mNPC is approximately 20% [3, 4]. According to the current National Comprehensive Cancer Network (NCCN) guidelines, platinum-based palliative chemotherapy (PCT) with or without locoregional radiotherapy (RT) is the cornerstone of treatment for patients with mNPC [5]. Many retrospective studies have asserted that cycles of PCT are not always positively related to survival and 4–6 cycles are recommended [6–8]. Moreover, the addition of RT has been shown to significantly benefit the prognosis of patient, increasing the 5-year median overall survival (OS) time to 21–36 months compared with 10–15 months for PCT alone [1, 9, 10]. The sequence and interval of PCT and RT have been explored, and no difference was found between concurrent chemoradiotherapy (CCRT) and PCT followed by RT, or RT initiation within 10 days or even 120 days after PCT [9, 10]. Recently, a phase III trial comparing the survival of patients with or without RT in endemic regions demonstrated that RT added to PCT significantly improved OS in chemotherapy-sensitive patients with mNPC (24-month OS, 76.4% vs 54.5%; hazard ratio [HR], 0.42 (0.23–0.77); P = 0.004) [11]. However, the details related to combining chemotherapy and radiotherapy, such as whether concurrent chemotherapy is still necessary after PCT or whether the number of PCT cycles can be decreased when RT is applied, remain unclear. Thus, this study aimed to evaluate survival outcomes and identify a promising strategy for mNPC patients treated with PCT and RT.

## Methods

### Study population

Consecutive patients with de novo metastatic nasopharyngeal carcinoma diagnosed at the Sun Yat-sen University Cancer Center between January 1, 2000, and December 31, 2017, were reviewed retrospectively. The eligibility criteria for this study were as follows: (1) biopsy-proven NPC according to the pathological classification system of the World Health Organization (WHO); (2) pathologically or radiologically confirmed distant metastasis at initial diagnosis; (3) treated with PCT followed by RT with or without concurrent chemotherapy; and (4) the absence of other malignant diseases. Patients were excluded if their clinical data were missing, such as the tumor category, node category or metastatic sites. The study cohort ultimately included 355 patients (Additional file 1: Fig. S1).

The study was approved by the Institutional Review Board of Sun Yat-sen University Cancer Center, and informed consent was waived. The study was performed in accordance with institutional policy to protect patients’ private information. The authenticity of this article has been validated by uploading the key raw data onto the Research Data Deposit public platform (www.researchdata.org.cn).

### Treatment

All eligible patients received at least one cycle of the following chemotherapy regimens before RT: cisplatin plus 5-fluorouracil (PF, 80 mg/m^2^ cisplatin intravenously [IV] on day 1 plus 1000 mg/m^2^/day 5-fluorouracil continuous IV infusion on days 1–4), cisplatin plus docetaxel (TP, 75 mg/m^2^ cisplatin IV on day 1 plus 75 mg/m^2^ docetaxel IV on day 1), cisplatin plus docetaxel plus 5-fluorouracil (TPF, 75 mg/m^2^ cisplatin IV on day 1 plus 75 mg/m^2^ docetaxel IV on day 1 plus 750 mg/m^2^/day 5-fluorouracil continuous IV infusion on days 1–5), or others. All patients underwent RT with two- or three-dimensional conventional radiotherapy, or intensity-modulated radiotherapy (IMRT) on a conventional schedule (5 daily fractions per week). Prescribed doses complied with our center’s guidelines with 66–70 Gy for gross tumor volume (GTVnx), 64–70 Gy for the involved cervical lymph nodes (GTVnd), 60–62 Gy for high-risk clinical target volume (CTV1), and 54–56 Gy for low-risk clinical target volume (CTV2) in 30–33 fractions. More details about chemotherapy and radiotherapy were provided in the Supplement (Additional file 1: Description of the radiotherapy and Table S1). The timing and combination of chemotherapy and radiotherapy were determined by the clinicians, patient’s condition and willingness. Twenty-one days to the commencement of radiotherapy from the end of the last chemotherapy cycle could be considered [11]. Local therapy for metastatic lesions, such as radiotherapy, surgery, transcatheter hepatic artery chemoembolization or radiofrequency ablation, was performed during treatment. The evaluation of tumor response to therapy was based on head and neck magnetic resonance imaging with contrast, chest radiography/chest computed tomography, abdominal sonography/abdominal computed tomography, bone scan or positron emission tomography/computed tomography, and patients were classified as having a complete response (CR), a partial response (PR), stable disease (SD), or progressive disease (PD) according to the Response Evaluation Criteria in Solid Tumors version 1.1.

### Statistical analysis

Statistical analyses were carried out with Statistical Product and Service Solutions software version 24.0 (SPSS Inc., Chicago, IL, USA) and R Statistical Software version 3.2.0 (Foundation for Statistical Computing, Vienna, Austria). All analyses were two-tailed, and the significance level was specified as p < 0.05. The primary objective of this study was to compare the OS of patients with mNPC treated with RT with or without concurrent chemotherapy. OS was defined as the time from metastasis to death from any cause or censored at the last visit or the final follow-up date of December 31, 2019. The secondary objective was progression-free survival (PFS), which was defined as the time from metastasis to disease progression or death from any cause or censored at the last visit or the final follow-up date of December 31, 2019. More specifically, locoregional progression-free survival (LRPFS) and distant progression-free survival (DPFS) were analyzed. The chi-square test or Fisher's exact test was used to compare categorical variables between two groups. Then, the baseline characteristics with significant differences were introduced into the logistic regression model to compute a propensity score for every patient. Propensity score matching (PSM) was employed to match patients with or without concurrent chemotherapy using the 1:1 nearest neighbor technique with a caliper of 0.1 to ensure a relatively good balance. Survival curves were evaluated using the Kaplan–Meier method with the log-rank test. Cox proportional hazards modeling was used to determine prognostic factors for OS. Multivariate analysis via stepwise selection was conducted including variables with P < 0.1 in univariate analyses. Exploratory subgroup analyses and landmark analyses were applied to the matched cohort to address bias from patients with a poor prognosis.

## Results

### Baseline characteristics

Among the 355 patients included in this study, 163 (45.9%) received CCRT, while 192 (54.1%) received RT alone. Patients and treatment characteristics before matching are summarized in Table 1. Age was divided into two groups by a median age of 45 years old. The use of CCRT increased as the year of diagnosis increased and was associated with T4 disease and the application of IMRT. Before RT, most patients received platinum-based chemotherapy, such as PF, TP, or TPF, and approximately 70% of them underwent 4–6 cycles. The median cycle number of PCT was 5 cycles in the PCT + CCRT group and 6 cycles in the PCT + RT group. The tumor response to PCT was satisfactory, and the most common response was PR. Even though patients received PCT, RT was still tolerated, as most patients had a Karnofsky performance score (KPS) rating of 90. The RT dose for more than 90% of the patients reached 60 Gy and their median doses were both 70 Gy. In addition to PCT and RT, 125 patients underwent local treatment at metastatic sites. Propensity score analysis was performed with the significant variables, including diagnosis year, T category, RT technique and RT dose, and all covariates were well balanced after matching with P > 0.1 (Table 1).

**Table 1:** Baseline characteristics

	Before matching			After matching		
	PCT + RT (N = 192) No. (%)	PCT + CCRT (N = 163) No. (%)	P-value	PCT + RT (N = 134) No. (%)	PCT + CCRT (N = 134) No. (%)	P-value
Age (years)						
< 45	102 (53.1)	81 (49.7)	0.590	75 (56.0)	69 (51.5)	0.462
≥ 45	90 (46.9)	82 (50.3)		59 (44.0)	65 (48.5)	
Sex						
Male	163 (84.9)	141 (86.5)	0.781	112 (83.6)	117 (87.3)	0.386
Female	29 (15.1)	22 (13.5)		22 (16.4)	17 (12.7)	
Diagnosis period						
2000–2005	26 (13.5)	12 (7.4)	0.030	14 (10.4)	12 (9.0)	0.865
2006–2011	66 (34.4)	45 (27.6)		33 (24.6)	36 (26.9)	
2012–2017	100 (52.1)	106 (65.0)		87 (64.9)	86 (64.2)	
Pathology						
I-II	22 (11.5)	13 (8.0)	0.358	12 (9.0)	10 (7.5)	0.656
III	170 (88.5)	150 (92.0)		122 (91.0)	124 (92.5)	
Tumor category						
T1	7 (3.6)	8 (4.9)	0.012	6 (4.5)	2 (1.5)	0.539
T2	33 (17.2)	13 (8.0)		15 (11.2)	13 (9.7)	
T3	95 (49.5)	72 (44.2)		65 (48.5)	67 (50.0)	
T4	57 (29.7)	70 (42.9)		48 (35.8)	52 (38.8)	
Node category						
N0	6 (3.1)	5 (3.1)	0.830	3 (2.2)	5 (3.7)	0.231
N1	39 (20.3)	31 (19.0)		24 (17.9)	28 (20.9)	
N2	79 (41.1)	75 (46.0)		50 (37.3)	60 (44.8)	
N3	68 (35.4)	52 (31.9)		57 (42.5)	41 (30.6)	
Bone metastasis						
Absent	47 (24.5)	49 (30.1)	0.150	34 (25.4)	41 (30.6)	0.605
Single	56 (29.2)	55 (33.7)		44 (32.8)	43 (32.1)	
Multiple	89 (46.4)	59 (36.2)		56 (41.8)	50 (37.3)	
Liver metastasis						
Absent	154 (80.2)	128 (78.5)	0.915	114 (85.1)	104 (77.6)	0.264
Single	17 (8.9)	15 (9.2)		8 (6.0)	14 (10.4)	
Multiple	21 (10.9)	20 (12.3)		12 (9.0)	16 (11.9)	
Lung metastasis						
Absent	165 (85.9)	128 (78.5)	0.148	108 (80.6)	104 (77.6)	0.832
Single	10 (5.2)	16 (9.8)		11 (8.2)	13 (9.7)	
Multiple	17 (8.9)	19 (11.7)		15 (11.2)	17 (12.7)	
No. of metastatic organs						
None	10 (5.2)	7 (4.3)	0.441	8 (6.0)	5 (3.7)	0.375
Single organ	157 (81.8)	127 (77.9)		109 (81.3)	105 (78.4)	
Multiple organs	25 (13.0)	29 (17.8)		17 (12.7)	24 (17.9)	
Sites of organ metastasis						
Absent	10 (5.2)	7 (4.3)	0.226	8 (6.0)	5 (3.7)	0.527
Single	62 (32.3)	67 (41.1)		47 (35.1)	54 (40.3)	
Multiple	120 (62.5)	89 (54.6)		79 (59.0)	75 (56.0)	
Distant nodal metastasis						
Absent	170 (88.5)	141 (86.5)	0.806	116 (86.6)	115 (85.8)	1.000
Single region	19 (9.9)	19 (11.7)		16 (11.9)	16 (11.9)	
Multiple regions	3 (1.6)	3 (1.8)		2 (1.5)	3 (2.2)	
Metastatic type						
Distant metastasis without LN involvement	170 (88.5)	141 (86.5)	0.547	116 (86.6)	115 (85.8)	0.506
Only distant LN metastasis	10 (5.2)	7 (4.3)		8 (6.0)	5 (3.7)	
Distant metastasis with LN involvement	12 (6.2)	15 (9.2)		10 (7.5)	14 (10.4)	
No. of metastatic lesions						
Oligo	60 (31.2)	68 (41.7)	0.053	45 (33.6)	53 (39.6)	0.310
Multiple	132 (68.8)	95 (58.3)		89 (66.4)	81 (60.4)	
PCT regimen						
PF	69 (35.9)	43 (26.4)	0.052	48 (35.8)	36 (26.9)	0.157
GP	12 (6.2)	4 (2.5)		7 (5.2)	4 (3.0)	
TP	46 (24.0)	39 (23.9)		35 (26.1)	31 (23.1)	
TPF	51 (26.6)	59 (36.2)		36 (26.9)	48 (35.8)	
Others	14 (7.3)	18 (11.0)		8 (6.0)	15 (11.2)	
No. of PCT cycles						
1–3	35 (18.2)	41 (25.2)	0.232	23 (17.2)	33 (24.6)	0.317
4–6	142 (74.0)	113 (69.3)		103 (76.9)	93 (69.4)	
> 6	15 (7.8)	9 (5.5)		8 (6.0)	8 (6.0)	
Response to PCT						
CR	6 (3.1)	3 (1.8)	0.214	5 (3.7)	3 (1.8)	0.282
PR	123 (64.1)	96 (58.9)		86 (64.2)	78 (58.2)	
SD	18 (9.4)	27 (16.6)		12 (9.0)	22 (16.4)	
Unknown	45 (23.4)	37 (22.7)		31 (23.1)	31 (23.1)	
KPS before RT						
90	159 (82.8)	143 (87.7)	0.147	113 (84.3)	118 (88.1)	0.227
80	9 (4.7)	10 (6.1)		7 (5.2)	9 (6.7)	
70	2 (1.0)	2 (1.2)		1 (0.7)	2 (1.5)	
Unknown	22 (11.5)	8 (4.9)		13 (9.7)	5 (3.7)	
Technique of RT						
Conventional RT	67 (34.9)	28 (17.2)	< 0.001	29 (21.6)	28 (17.2)	1.000
IMRT	113 (58.9)	131 (80.4)		104 (77.6)	104 (77.6)	
Unknown	12 (6.2)	4 (2.5)		1 (0.7)	2 (1.5)	
RT dose (Gy)						
< 60	11 (5.7)	2 (1.2)	0.020	1 (0.7)	2 (1.5)	1.000
≥ 60	179 (93.2)	161 (98.8)		133 (99.3)	132 (98.5)	
Unknown	2 (1.0)	0 (0.0)		0 (0.0)	0 (0.0)	
Local treatment of metastatic sites						
No	128 (66.7)	102 (62.6)	0.489	81 (60.4)	86 (64.2)	0.529
Yes	64 (33.3)	61 (37.4)		53 (39.6)	48 (35.8)	

*PCT* palliative chemotherapy, *RT* radiotherapy, *CCRT* concurrent chemoradiotherapy, *No.* number, *LN* lymph node, *PF* cisplatin plus 5-fluorouracil, *GP* gemcitabine plus cisplatin, *TP* cisplatin plus docetaxel, *TPF* cisplatin plus docetaxel plus 5-fluorouracil, *CR* complete response, *PR* partial response, *SD* stable disease, *KPS* Karnofsky performance score, *IMRT* intensity-modulated radiotherapy

### Survival outcomes of CCRT after PCT

**Fig. 1:**
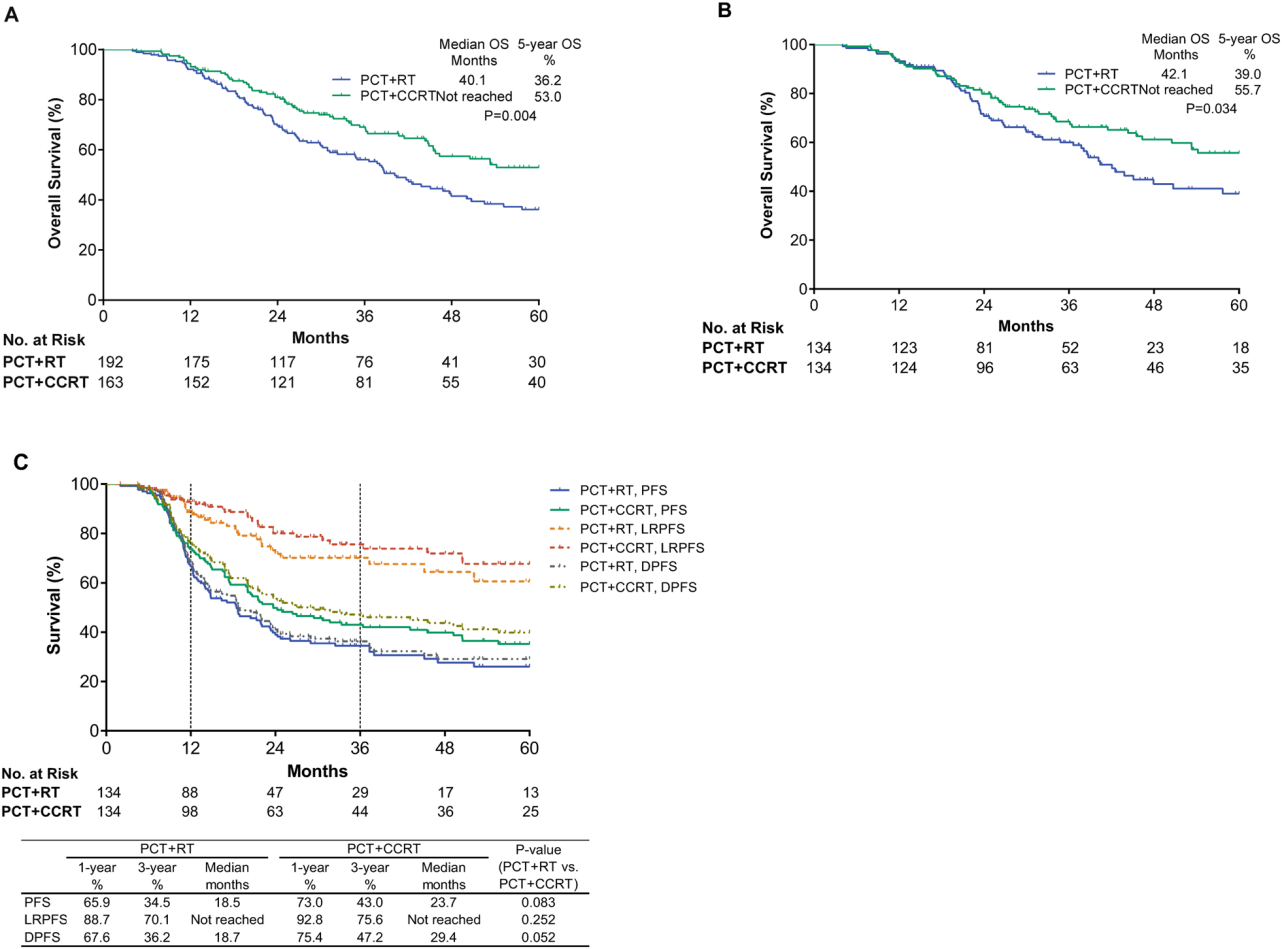
Survival outcomes for patients treated with CCRT or RT after PCT. **A** OS for all patients. **B** OS for propensity score-matched patients. **C** PFS, including LRPFS and DPFS for propensity score-matched patients. *CCRT* concurrent chemoradiotherapy, *RT* radiotherapy, *PCT* palliative chemotherapy, *OS* overall survival, *PFS* progression-free survival, *LRPFS* locoregional progression-free survival, *DPFS* distant progression-free survival

The median follow-up time for the entire cohort was 50.8 months (95% confidence interval [CI], 45.0–56.6 months). The addition of concurrent chemotherapy significantly improved survival outcomes, with a median OS time out of 60 months and a 5-year OS rate of 53.0% compared to 40.1 months and 36.2%, respectively, for RT alone (P = 0.004) (Fig. 1A). In the matched cohort of 268 patients, similar results were observed, with median OS times out of 60 months vs 42.1 months and 5-year OS rates of 55.7% vs 39.0%, respectively (P = 0.034) (Fig. 1B). PFS was also analyzed in our matched cohort (Fig. 1C), but no significant difference between the two groups was observed (median PFS, 18.5 vs 23.7 months; P = 0.083). However, the difference in DPFS between the PCT + CCRT group and the PCT + RT group was close to being statistically significant (median DPFS, 29.4 vs 18.7 months; P = 0.052).

### Prognostic factors

The results of the univariate and multivariate Cox regression models in the matched cohort were summarized in Table 2. As only 13 patients had lymph node metastasis without organ metastasis, 11 patients had GP regimen, and 3 patients received RT with a maximum dose less than 60 Gy, these patients were excluded in the analyses. On univariate analysis, the addition of CCRT to PCT, later diagnosis, 4–6 cycles of PCT before RT and local treatment at metastatic sites were associated with longer OS, while multiple liver metastases or multiple metastatic lesions were correlated with diminished OS. Ten variables with P < 0.1 in univariate analyses were entered into multivariate analysis, and PCT + CCRT remained independently associated with improved OS (HR, 0.59; 95% CI, 0.39–0.88; P = 0.009). Additional favorable prognostic factors for OS included females and ≥ 4 cycles of PCT. Multiple liver metastases and multiple metastatic lesions were still independent risk factors for OS.

**Table 2:** Univariate and multivariate Cox regression models in the propensity score-matched cohort

	Univariate		Multivariate	
	HR (95% CI)	P-value	HR (95% CI)	P-value
Group				
PCT + RT vs. PCT + CCRT	0.67 (0.46–0.97)	0.036	0.59 (0.39–0.88)	0.009
Age (years)				
< 45 vs. ≥ 45	1.18 (0.81–1.72)	0.389		
Sex				
Male vs. Female	0.53 (0.28–1.00)	0.054	0.50 (0.27–0.94)	0.033
Diagnosis period				
2000–2005 vs. 2006–2011	0.52 (0.30–0.92)	0.025		
2000–2005 vs. 2012–2017	0.46 (0.27–0.77)	0.003		
Pathology				
I-II vs. III	0.78 (0.58–1.04)	0.093		
Tumor category				
T1-2 vs. T3-4	1.06 (0.61–1.83)	0.834		
Node category				
N0-1 vs. N2-3	1.10 (0.71–1.71)	0.670		
Bone metastasis				
Absent vs. Single	0.66 (0.39–1.11)	0.117		
Absent vs. Multiple	1.77 (0.87–2.12)	0.177		
Liver metastasis				
Absent vs. Single	0.83 (0.40–1.71)	0.606	1.06 (0.51–2.22)	0.871
Absent vs. Multiple	2.32 (1.38–3.88)	0.001	1.95 (1.13–3.35)	0.016
Lung metastasis				
Absent vs. Single	0.79 (0.38–1.62)	0.514		
Absent vs. Multiple	1.32 (0.74–2.37)	0.346		
Distant nodal metastasis				
Absent vs. Present	1.53 (0.92–2.55)	0.100		
No. of metastatic organs				
Single organ vs. Multiple organs	1.62 (0.98–2.66)	0.059		
Metastatic situation				
Organ metastasis without LN involvement vs. Organ metastasis with LN involvement	1.26 (0.94–1.69)	0.116		
No. of metastatic lesions				
Oligo vs. Multiple	2.18 (1.42–3.34)	< 0.001	1.97 (1.23–3.16)	0.005
PCT regimen				
PF vs. TP	1.21 (0.77–1.91)	0.415		
PF vs. TPF	0.65 (0.40–1.05)	0.077		
No. of PCT cycles				
1–3 vs. 4–6	0.61 (0.40–0.92)	0.020	0.49 (0.31–0.77)	0.002
1–3 vs. > 6	0.47 (0.18–1.22)	0.120	0.27 (0.09–0.77)	0.014
Response to PCT				
CR/PR vs. SD	1.19 (0.93–1.53)	0.159		
KPS before RT				
90 vs. 70–80	1.30 (0.69–2.42)	0.417		
Technique of RT				
Conventional RT vs. IMRT	0.74 (0.49–1.13)	0.162		
Local treatment of metastatic sites				
No vs. Yes	0.65 (0.44–0.98)	0.040	0.69 (0.45–1.05)	0.082

*PCT* palliative chemotherapy, *RT* radiotherapy, *CCRT* concurrent chemoradiotherapy, *HR* hazard ratio, *CI* confidence interval, *No.* number, *LN* lymph node, *PF* cisplatin plus 5-fluorouracil, *TP* cisplatin plus docetaxel, *TPF* cisplatin plus docetaxel plus 5-fluorouracil, *CR* complete response, *PR* partial response, *SD* stable disease, *KPS* Karnofsky performance score, *IMRT* intensity-modulated radiotherapy

### Combination of PCT and RT

**Fig. 2:**
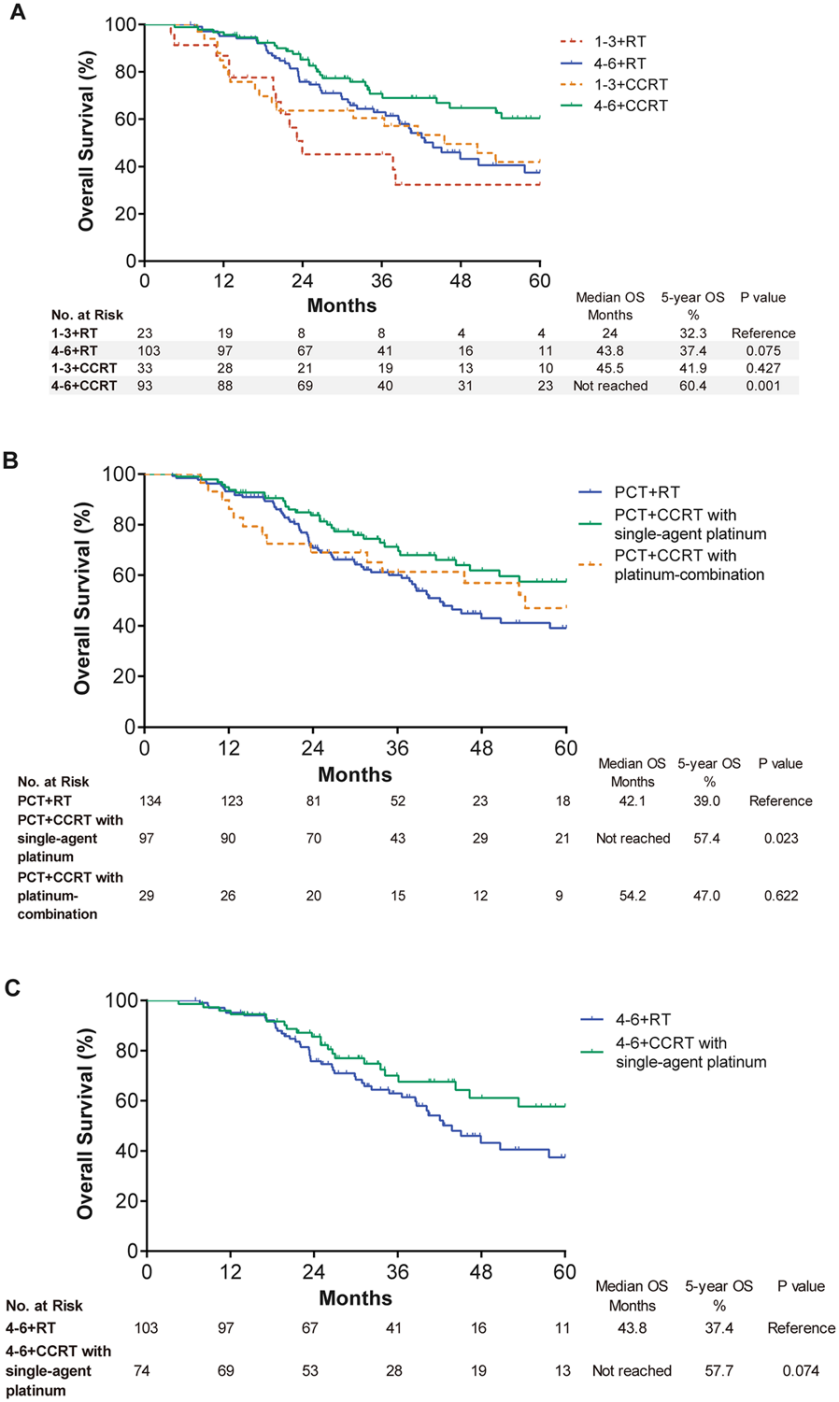
OS for patients receiving different combinations of systemic and locoregional treatments after propensity score matching. **A** Different cycles of PCT. **B** Different regimens of concurrent chemotherapy. **C** Single-agent platinum versus radiotherapy alone after 4–6 cycles of PCT. *OS* overall survival, *PCT* palliative chemotherapy, *CCRT* concurrent chemoradiotherapy, *RT* radiotherapy

To determine whether the addition of concurrent chemotherapy could reduce the intensity of PCT before RT, we analyzed survival outcomes with different combinations of PCT and RT for the matching patients, showing the results in Fig. 2. Regardless of the PCT + RT group or PCT + CCRT group, the prognosis with 4–6 cycles of PCT was better than that with 1–3 cycles of PCT, among which 4–6 cycles of PCT followed by CCRT had excellent survival with a 5-year OS rate of 60.4% (Fig. 2A). Therefore, enough cycles of PCT were necessary even with the addition of concurrent chemotherapy. Furthermore, we performed the analysis in patients with or without concurrent single-agent platinum such as cisplatin or platinum-combination chemotherapy such as PF. We identified that concurrent single-agent platinum was more appropriate during RT in patients with PCT (Fig. 2B). A trend toward improved OS was observed for patients receiving concurrent single-agent platinum after 4–6 cycles of PCT (P = 0.074) (Fig. 2C).

### Subgroup and landmark analyses

**Fig. 3:**
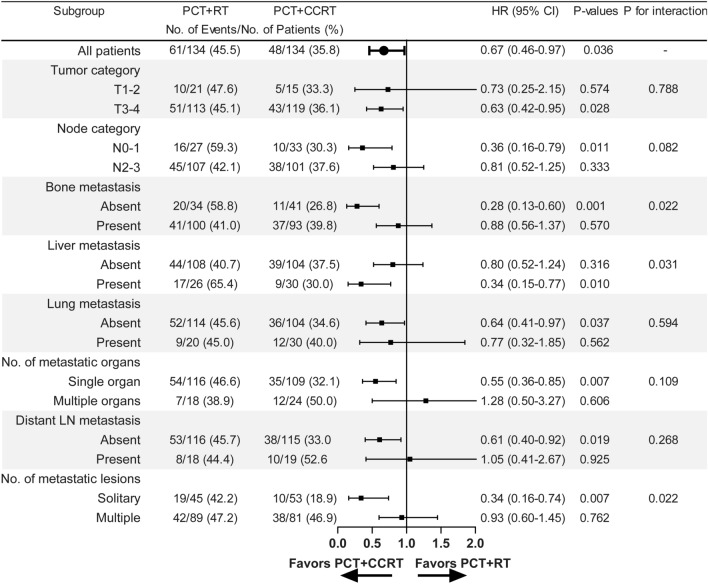
Forest plot of CCRT versus RT after PCT by subgroups for propensity score-matched patients. *CCRT* concurrent chemoradiotherapy, *RT* radiotherapy, *PCT* palliative chemotherapy, *HR* hazard ratio, *CI* confidence interval, *No.* number, *LN* lymph node

Subgroup analyses of clinical factors were shown in Fig. 3. The absence of bone metastasis, the presence of liver metastasis and a solitary metastasis derived more benefit from PCT followed by CCRT.

**Fig. 4:**
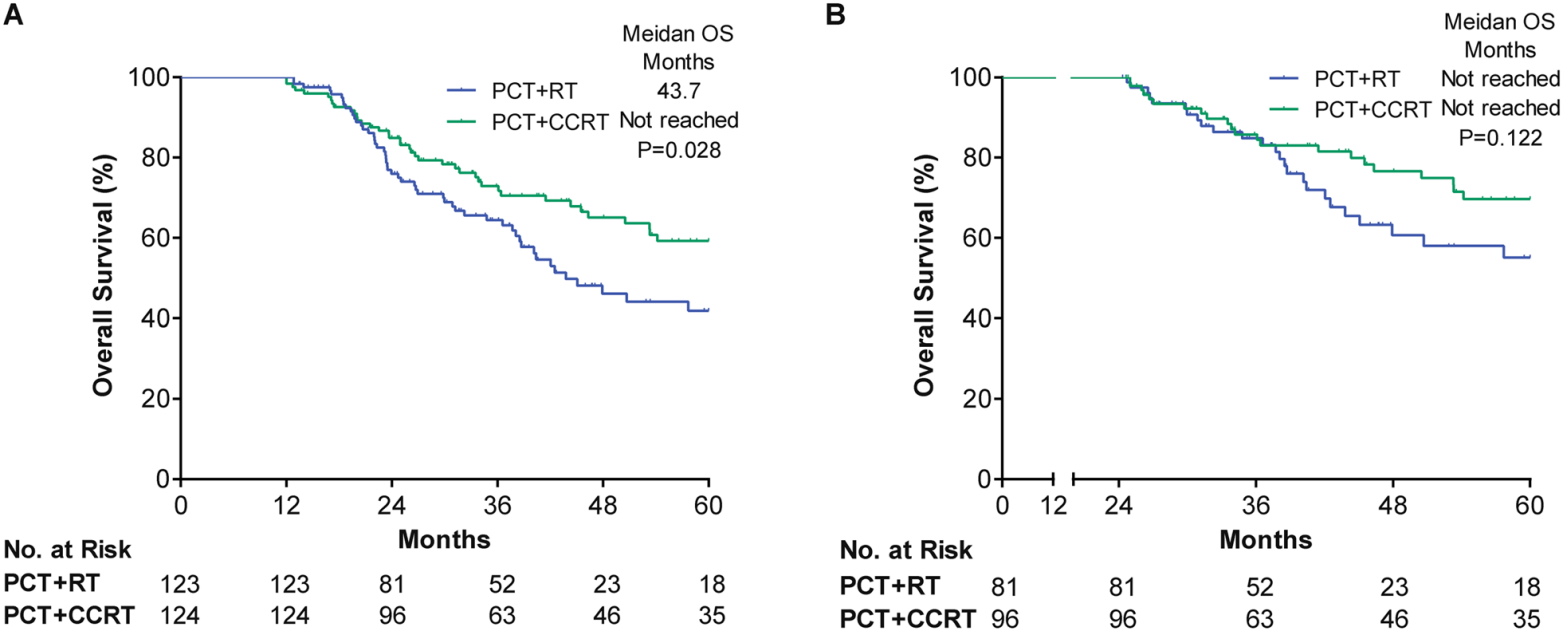
Landmark analysis of OS for propensity score-matched patients receiving PCT followed by CCRT or RT. **A** Patients survived for ≥ 1 year. **B** Patients survived for ≥ 2 years. *OS* overall survival; *PCT* palliative chemotherapy, *CCRT* concurrent chemoradiotherapy, *RT* radiotherapy

The results of landmark analysis, which evaluated the impact of CCRT after PCT on long-term survival, were presented in Fig. 4. Among the patients who survived for ≥ 1 year after diagnosis, PCT followed by CCRT remained associated with improved OS (P = 0.028). However, this survival difference was not significant in the patients who survived for ≥ 2 years (P = 0.122).

## Discussion

Benefits from the treatment of primary tumors in metastatic disease have been demonstrated for multiple tumor types, such as renal cancer, breast cancer and NPC [12, 13]. The OS of patients with mNPC who received RT was prolonged in our study (Additional file 1: Fig. S2), consistent with the results of previous studies. Although platinum-based chemotherapy remains the foundation of mNPC treatment, the phase III trial comparing the survival of patients with or without RT in endemic regions confirmed that radiotherapy added to chemotherapy significantly improved OS in chemotherapy-sensitive patients with mNPC (2-year OS, 76.4% vs 54.5%; P = 0.004), providing more convincing evidence [11]. However, the long-term efficacy of the trial has not yet been reported, and the 5-year OS rate of RT after PCT in previous retrospective studies had heterogeneous results varying from 16.6% to 34%, which was disappointing when compared with the 5-year OS of approximately 80% for non-metastatic NPC (Additional file 1: Table S2) [9, 10, 14–16]. What else can we do for mNPC except for the addition of RT? The purpose of this study was to provide a useful strategy for clinicians in treating mNPC with traditional therapies. To the best of our knowledge, this study is the first attempt to compare PCT followed by RT with or without concurrent chemotherapy.

In this study, we report the outcomes of continuous de novo metastatic NPC patients treated with PCT and RT at an institution in an endemic area for nearly 20 years. The addition of chemotherapy during RT significantly improved OS compared with RT alone when finishing PCT. An absolute improvement in the 5-year OS rate of approximately 16.8% was identified for the entire cohort. After addressing selection limitations related to the use of PSM, the advantage of CCRT could still not be ignored. The best time to perform CCRT was suggested to be after 4–6 cycles of PCT, and single-agent platinum therapeutics such as cisplatin can be of great benefit to patients.

Concurrent chemoradiotherapy has been recommended for patients with locoregionally advanced NPC [17]. A phase II randomized controlled trial demonstrated that concurrent weekly cisplatin chemotherapy could improve the OS of patients with locally recurrent NPC, especially those with disease classified in an advanced T category. The difference in grade 3–4 toxicity between CCRT and RT alone was not significant [18]. In other metastatic tumor types, CCRT was reported to improve survival outcomes [19–22]. In our study, CCRT was recommended as survival was significantly improved while most grade 3 to 4 toxic effects were not significantly increased (Additional file 1: Table S3). We believe that concurrent chemotherapy enhances tumor control, acts as a radiotherapy sensitizer and eradicates micrometastases, leading to an additive or synergistic effect on tumor killing. Additionally, control of the primary tumor prevents further self-seeding of metastases [23], and the abscopal effect of radiotherapy induces regression at nonirradiated, distant tumor sites [24].

The details for chemotherapy before or during RT, such as agents and cycle numbers, are inconclusive due to limited medical records [9]. Some evidence indicated that there was no significant difference between patients who received at least six cycles of chemotherapy and those who received less than six cycles [25]. Chemoresistance may occur as the number of cycles increases. However, progression may appear with less intense chemotherapy, especially in patients who undergo fewer than four cycles [6, 7]. After patients receive 4–6 cycles of chemotherapy, chemoradiotherapy should be applied. A single agent for concurrent chemotherapy, such as cisplatin, may be the best choice because it has less severe side effects, unlike doublet or multiple agents, and much evidence has demonstrated the important role of cisplatin in concurrent chemotherapy [26].

The prognostic characteristics of mNPC have been explored in many studies. A number of these studies have indicated that patients with limited metastatic lesions had more favorable outcomes than those with liver metastasis or multiple metastatic lesions [27, 28]. For patients with favorable outcomes, consolidated therapy of the primary tumor may make metastatic disease a curative disease, while actively curing the primary disease may prolong local control for patients with unfavorable outcomes. However, identifying candidates who are most likely to benefit from CCRT needs further research. Relatively good outcomes have been observed for patients receiving RT if they were sensitive to PCT or if post-PCT Epstein-Barr virus (EBV) levels decreased [29, 30]. Perhaps it helps when selecting patients to deliver CCRT.

Currently, there is an exciting era of developing immune checkpoint inhibitors in NPC [31]. A phase I clinical trial of 27 patients with recurrent or metastatic NPC suggested that pembrolizumab treatment resulted in a median OS of 16.5 months. [32] Another phase II trial of nivolumab also suggested the potential use of immunotherapy for mNPC as the median OS of 44 patients receiving nivolumab was 17.1 months. [33] In addition to monotherapy, a phase I clinical trial from China demonstrated that the therapeutic effect of camrelizumab combined with chemotherapy was superior to that of camrelizumab alone [34]. The superior efficacy of the camrelizumab combination was recently confirmed in a phase III randomized study [35]. Therefore, how to use immunotherapy in addition to chemotherapy and radiotherapy to maximize the survival of patients with mNPC is worth further effort.

Our study had several limitations that should be mentioned. The source of patients who underwent PCT followed by RT was restricted to one hospital, and the sample size was not sufficiently large. As this study was retrospective in nature, selection bias and imbalances existed. Plasma EBV testing results were not available, although EBV was an important factor for therapeutic monitoring and prognostic evaluations. In addition, quality of life, late toxicity and some details on the following lines of therapy were not considered in this study. Thus, prospective studies are warranted to support our findings.

## Conclusions

The real-world study suggests that concurrent chemoradiotherapy significantly improves OS compared with radiotherapy alone after palliative chemotherapy in patients with de novo metastatic nasopharyngeal carcinoma. More specifically, concurrent chemoradiotherapy with single-agent platinum after 4–6 cycles of chemotherapy can be considered.

## Supplementary Information

**Additional file 1: Description of the radiotherapy. Figure S1.** Patient selection diagram.** Figure S2.** Overall Survival (OS) for patients treated with chemoradiotherapy (CRT) or palliative chemotherapy (PCT) in de novo metastatic nasopharyngeal carcinoma. **Table S1. **Details of common chemotherapy regimens. **Table S2.** Summary of studies related to locoregional radiotherapy in de novo metastatic nasopharyngeal carcinoma. **Table S3.** Adverse effects.** References.**

## Data Availability

The authenticity of this article has been validated by uploading the key raw data onto the Research Data Deposit public platform (www.researchdata.org.cn). All data will be shared upon request to the corresponding author.

## Abbreviations

NPC: Nasopharyngeal carcinoma
mNPC: De novo metastatic nasopharyngeal carcinoma
NCCN: National Comprehensive Cancer Network
PCT: Palliative chemotherapy
RT: Radiotherapy
OS: Overall survival
CCRT: Concurrent chemoradiotherapy
HR: Hazard ratio
WHO: World Health Organization
PF: Cisplatin plus 5-fluorouracil
TP: Cisplatin plus docetaxel
TPF: Cisplatin plus docetaxel plus 5-fluorouracil
IMRT: Intensity-modulated radiotherapy
CR: Complete response
PR: Partial response
SD: Stable disease
PD: Progressive disease
PFS: Progression-free survival
LRPFS: Locoregional progression-free survival
DPFS: Distant progression-free survival
PSM: Propensity score matching
CI: Confidence interval
KPS: Karnofsky performance score
EBV: Epstein-Barr virus
