# Supplementary material for: Addition of chemoradiotherapy to palliative chemotherapy in de novo metastatic nasopharyngeal carcinoma: a real-world study
Source: Cancer Cell Int. 2022 Jan 24;22:36. doi: 10.1186/s12935-022-02464-7 (PMC8788066; doi:10.1186/s12935-022-02464-7)
Supplement: Supplementary file 1 — Additional file 1: Description of the radiotherapy. Figure S1. Patient selection diagram. Figure S2. Overall Survival (OS) for patients treated with chemoradiotherapy (CRT) or palliative chemotherapy (PCT) in de novo metastatic nasopharyngeal carcinoma. Table S1. Details of common chemotherapy regimens. Table S2. Summary of studies related to locoregional radiotherapy in de novo metastatic nasopharyngeal carcinoma. Table S3. Adverse effects. References. [file 12935_2022_2464_MOESM1_ESM.docx]

**Additional file 1 Content**

**Description of the radiotherapy.**

**Figure S1.** Patient selection diagram

**Figure S2.** Overall Survival (OS) for patients treated with chemoradiotherapy (CRT) or palliative chemotherapy (PCT) in de novo metastatic nasopharyngeal carcinoma

**Table S1.** Details of Common Chemotherapy Regimens

**Table S2.** Summary of studies related to locoregional radiotherapy in de novo metastatic nasopharyngeal carcinoma

**Table S3.** Adverse Effects

**References.**

**Description of the radiotherapy**

In general, all patients were immobilized in the supine position and using a thermoplastic mask that covered the head, neck, and shoulder. Both non-enhanced CT (for dose calculation) and contrastenhanced CT (for target delineation) images were obtained from the vertex to 2 cm below the sternoclavicular joint, with 3-mm slices.

Target volumes were defined in accordance with the International Commission on Radiation Units and Measurements (ICRU) reports 50 and 62. Gross tumor volume (GTV) was defined as the gross tumor determined by physical examination, imaging (including MRI and PET/CT, if available) and endoscopic findings before induction chemotherapy or concurrent chemoradiotherapy, including GTVnx and GTVnd. GTVnx included the sum of the primary tumor volume and the enlarged retropharyngeal nodes, while GTVnd was the volume of involved gross cervical lymph nodes. The high-risk clinical target volume (CTV1) was defined as the GTVnx plus a 5–10-mm margin (2–3 mm posteriorly if adjacent to the brain stem or spinal cord) to encompass the high-risk sites of microscopic extension and the whole nasopharynx. The low-risk clinical target volume (CTV2) was defined as the CTV1 plus a 5–10-mm margin (2–3 mm posteriorly if adjacent to the brain stem or spinal cord) to encompass the low-risk sites of microscopic extension, including the clivus, sphenoid sinus, foramen lacerum, oval foramen, parapharyngeal space, pterygoid fossae, posterior parts of the nasal cavity, pterygopalatine fossae, retropharyngeal nodal regions, the cervical level where the involved lymph nodes were located, the elective neck area from level II to Vb, and the supraclavicular fossae. Level Ib was electively irradiated if: (1) Level Ib lymph nodes (LNs) were involved, (2) level IIa LNs had extracapsular extension or a diameter ≥ 3 cm, (3) there was extensive nodal disease on the ipsilateral neck, and (4) the soft or hard palate, oral cavity, or ipsilateral nasal cavity were grossly involved. A planning target volume (PTV) was created by adding a three-dimensional margin of 3–5 mm to the delineated target volume to compensate for the uncertainties in treatment set-up and internal organ motion. A 3-mm margin was added to the critical organs (e.g., brainstem and spinal cord) to form the planning organ at risk volume (PRV).

The prescribed doses were 66–70 Gy, 64–70 Gy, 60–62 Gy, and 54–56 Gy, in 30–33 fractions, for the PTVs derived from GTVnx, GTVnd, CTV1, and CTV2, respectively. The normal tissue dose constraints are listed in Table below. All plans were generated by a team of dosimetrists using a wholefield (including neck radiation) simultaneous integrated boost technique. In general, when criticalnormal tissues (e.g., brain stem and spinal cord) were adjacent to the high-dose target volumes, the target volume coverage could be compromised to keep these critical normal tissues within the dose constraints. When other normal tissues of lower priority were adjacent to the high-dose target volumes, the dose to these tissues was kept as low as possible without compromising the target coverage. The trade-off between covering the target volume and protecting normal tissue in each case was discussed and decided upon by the research team at each institution. Radiation was delivered once daily at five fractions per week.

**Normal tissue dose constraints used for plan optimization**

| **Structure** | **Dose constraints** |
| --- | --- |
| Spinal cord | Dmax* ≤ 45 Gy |
| Spinal cord_PRV | D1† ≤ 50 Gy |
| Brain stem | Dmax ≤ 54 Gy |
| Brain stem_PRV | D1 ≤ 60 Gy |
| Optic nerves | Dmax ≤ 54 Gy |
| Optic nerves_PRV | D1 ≤ 60 Gy |
| Optic chiasm | Dmax ≤ 54 Gy |
| Optic chiasm_PRV | D1 ≤ 60 Gy |
| Temporal lobe | Dmax ≤ 60 Gy |
| Temporal lobe_PRV | D1 ≤ 65 Gy |
| Lens | Dmean‡ < 8 Gy |
| Pituitary | Dmax < 60 Gy |
| Eyes | Dmean < 35 Gy |
| Mandible | Dmax < 70 Gy |
| Temporomandibular Joint | Dmax < 70 Gy |
| Parotid | Dmean < 26 Gy |
|  | V30§ < 50% |
| Cochlea | Dmean < 50 Gy |
| Larynx | Dmean < 45 Gy |

PRV = planning organ at risk volume.

* Maximum point dose to the target volume.

† Dose received by 1% of the target volume.

‡ Mean dose to the target volume.

§ At least 50% of the gland will receive <30 Gy (should be achieved in at least one gland).

**Figure S1.** Patient selection diagram

Patients with newly diagnosed metastatic NPC from 2000-2017 at SYSUCC (n=746)

Exclude (n=33)

Missing information

Patients with complete information (n=713)

Exclude (n=358)

● Chemotherapy alone (324)

● Without chemotherapy before RT (34)

Patients with PCT followed by RT(n=355)

● PCT plus RT alone (192)

● PCT plus CCRT (163)

Patients with treatment of metastatic sites (n=125)

Patients without treatment of metastatic sites (n=230)

Abbreviations: NPC, nasopharyngeal carcinoma; SYSUCC, Sun Yat-sen University Cancer Center; PCT, palliative chemotherapy; RT, radiotherapy; CCRT, concurrent chemoradiotherapy.

**
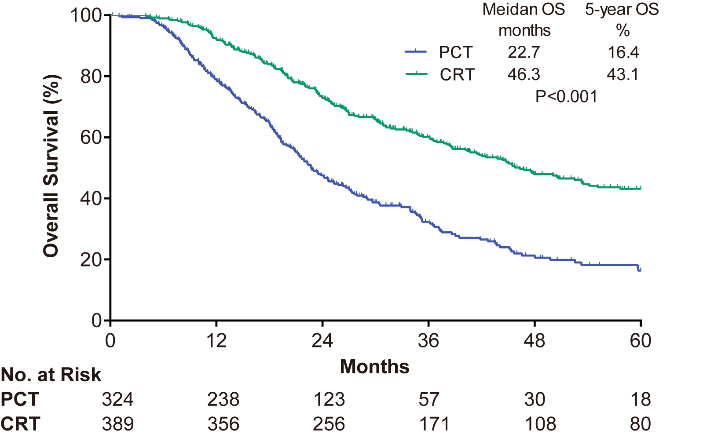
**

**Figure S2.** Overall Survival (OS) for patients treated with chemoradiotherapy (CRT) or palliative chemotherapy (PCT) in de novo metastatic nasopharyngeal carcinoma

**Table S1.** Details of Common Chemotherapy Regimens

| **Regimen** | **Drug** | **Dose** | **Day** | **Route** | **Cycle Frequency** |
| --- | --- | --- | --- | --- | --- |
| GP | gemcitabine | 1,000 mg/m^2^ | 1, 8 | IV infusion | 21 days |
|  | cisplatin | 80 mg/m^2^ | 1 | IV infusion |  |
| PF | 5-fluorouracil | 1,000 mg/m^2^/day | 1-4 | continuous IV infusion | 21 days |
|  | cisplatin | 80 mg/m^2^ | 1 | IV infusion |  |
| TP | docetaxel | 75 mg/m^2^ | 1 | IV infusion | 21 days |
|  | cisplatin | 75 mg/m^2^ | 1 | IV infusion |  |
| TPF | docetaxel | 60~75 mg/m^2^ | 1 | IV infusion | 21 days |
|  | cisplatin | 60~75 mg/m^2^ | 1 | IV infusion |  |
|  | fluorouracil | 600~750 mg/m^2^/day | 1-5 | continuous IV infusion |  |
| Cisplatin |  | 40 mg/m^2^ or  100 mg/m^2^ | 1 | IV infusion | 7 days or  21 days |

Abbreviations: GP, cisplatin plus gemcitabine; IV, intravenous; PF, cisplatin plus 5-fluorouracil; TP, cisplatin plus taxane; TPF, cisplatin plus taxane plus fluorouracil.

**Table S2.** Summary of studies related to locoregional radiotherapy in de novo metastatic nasopharyngeal carcinoma

| Author  Journal  (Year) | Study | Treatment | No. | mOS  (mo) | OS rate at different years (%) | | | |
| --- | --- | --- | --- | --- | --- | --- | --- | --- |
|  |  |  |  |  | 1 | 2 | 3 | 5 |
| You et al^1^ | -China  -Multicenter  -Phase III  RCT | Chemo + LRRT | 63 |  | 93.6 | 76.4 |  |  |
| *JAMA Oncol*  (2020) |  | Chemo Alone | 63 |  | 81.9 | 54.5 |  |  |
| Rusthoven et al^2^ | -United States  -Multicenter registry (NCDB)  -Retro | Chemo Alone  Chemo + LRRT | 281  437 | 15.5  21.4 |  |  |  | 10  28 |
| *Radiother Oncol* |  |  |  |  |  |  |  |  |
| (2017) |  |  |  |  |  |  |  |  |
| Verma et al^3^ | -United States  -Multicenter registry (NCDB)  -Retro | Chemo Alone  Chemo + LRRT | 296  259 | 13.7  25.8 | 54  72 |  | 21  41 | 10  34 |
| *J Natl Compr Canc Netw* |  |  |  |  |  |  |  |  |
| (2017) |  |  |  |  |  |  |  |  |
| Yin et al^4^ | -China  -Single center  -Retro | Chemo + LRRT | 32 |  |  | 75.2 | 50.1 |  |
| *Oncotargets Ther*  (2017) |  |  |  |  |  |  |  |  |
| Hu et al^5^ | -China  -Single center  -Retro | Chemo + LRRT | 41 | 31 | 89.9 | 67.4 | 41.1 | 22.5 |
| *Med Oncol*  (2015) |  |  |  |  |  |  |  |  |
| Zeng et al^6^ | -China  -Single center  -Retro | Chemo + LRRT  Chemo Alone | 140  94 |  |  |  | 48  12 |  |
| *PLoS One*  (2014) |  |  |  |  |  |  |  |  |
| Chen et al^7^ | -China  -Single center  -Retro | Chemo Alone  Chemo + LRRT  LRRT Alone  Best Supportive Care | 169  176  38  25 | 26  48  25  15 |  |  |  |  |
| *Chin J Cancer* |  |  |  |  |  |  |  |  |
| (2013) |  |  |  |  |  |  |  |  |
| Lin S et al^8^ | -China  -Single center  -Retro | Chemo + LRRT | 105 | 25 | 67.5 | 50.3 | 37.0 | 16.6 |
| *Am J Clin Oncol*  (2012) |  |  |  |  |  |  |  |  |
| Yeh et al^9^ | -Taiwan  -Single center  -Retro | LRRT Alone  Chemo Alone  Best Supportive Care | 58  39  28 |  | 48  36  25 | 24  10  0 |  |  |
| *Jpn J Clin Oncol*  (2005) |  |  |  |  |  |  |  |  |

Abbreviations: No., number; mOS, median overall survival; mo, months; OS, overall survival; RCT, randomized clinical trial; Chemo, chemotherapy; LRRT, locoregional radiotherapy; NCDB, National Cancer Database; Retro, retrospective study.

**Table S3.** Adverse Effects

|  | PCT+RT (N=192)  No. (%) | | |  | PCT+CCRT (N=163)  No. (%) | | | P for difference in all grades | P for difference in grade 3-4 |
| --- | --- | --- | --- | --- | --- | --- | --- | --- | --- |
| Event* | all grades | grade 3 | grade 4 |  | all grades | grade 3 | grade 4 |  |  |
| Acute hematologic toxicity | 184 (98.9) | 81 (43.5) | 26 (14.0) |  | 153 (96.2) | 45 (28.3) | 39 (24.5) | 0.150 | 0.382 |
| Leukopenia | 166 (89.2) | 41 (22.0) | 5 (2.7) |  | 145 (91.2) | 54 (34.0) | 7 (4.4) | 0.545 | **0.006** |
| Neutropenia | 158 (84.9) | 70 (37.6) | 17 (9.1) |  | 137 (86.2) | 39 (24.5) | 27 (17.0) | 0.749 | 0.327 |
| Anemia | 162 (87.1) | 20 (10.8) | 3 (1.6) |  | 139 (87.4) | 18 (11.3) | 12 (7.5) | 0.928 | 0.095 |
| Thrombocytopenia | 75 (40.3) | 14 (7.5) | 9 (4.8) |  | 78 (49.1) | 13 (8.2) | 10 (6.3) | 0.104 | 0.567 |
| Acute gastrointestinal toxicity | 90 (48.4) | 5 (2.7) | 0 |  | 111 (69.8) | 5(3.1) | 0 | **<0.001** | 1.000 |
| Acute hepatic toxicity | 65 (34.9) | 4 (2.2) | 1(0.5) |  | 39 (24.5) | 2 (1.3) | 0 | **0.036** | 0.459 |
| Acute nephritic toxicity | 35 (18.8) | 1(0.5) | 0 |  | 35 (22.0) | 0 | 0 | 0.462 | 1.000 |
| Acute toxicity specific to radiotherapy | 99 (52.4) | 16 (8.5) | 0 |  | 96 (59.6) | 24 (14.9) | 0 | 0.174 | 0.065 |
| Dry mouth | 73 (38.6) | 0 | 0 |  | 62 (38.5) | 0 | 0 | 0.982 | NA |
| Mucositis | 74 (39.2) | 14 (7.4) | 0 |  | 87 (54.0) | 23 (14.3) | 0 | **0.005** | **0.037** |
| Skin reaction | 66 (34.9) | 4 (2.1) | 0 |  | 67 (41.6) | 4 (2.5) | 0 | 0.198 | 1.000 |

Abbreviations: PCT, palliative chemotherapy; RT, radiotherapy; CCRT, concurrent chemoradiotherapy; No. number; NA, not applicable.

* Patients without records of events were excluded.

**References**

1. You R, Liu YP, Huang PY, et al. Efficacy and Safety of Locoregional Radiotherapy With Chemotherapy vs Chemotherapy Alone in De Novo Metastatic Nasopharyngeal Carcinoma. *JAMA Oncol* 2020;6(9):1345–1352.

2. Rusthoven CG, Lanning RM, Jones BL, et al. Metastatic nasopharyngeal carcinoma: Patterns of care and survival for patients receiving chemotherapy with and without local radiotherapy. Radiother Oncol 2017;124(1):139-146.

3. Verma V, Allen PK, Simone CN, et al. Addition of Definitive Radiotherapy to Chemotherapy in Patients With Newly Diagnosed Metastatic Nasopharyngeal Cancer. *J Natl Compr Canc Netw* 2017;15(11):1383-1391.

4. Yin ZZ, Zhang XM, Wang YY, et al. The combination of systemic therapy and locoregional radiotherapy prolongs survival in newly diagnosed metastatic nasopharyngeal carcinoma patients. *Oncotargets Ther* 2017;10:5677-5683.

5. Hu SX, He XH, Dong M, et al. Systemic chemotherapy followed by locoregional definitive intensity-modulated radiation therapy yields prolonged survival in nasopharyngeal carcinoma patients with distant metastasis at initial diagnosis. *Med Oncol* 2015;32(9)

6. Zeng L, Tian YM, Huang Y, et al. Retrospective analysis of 234 nasopharyngeal carcinoma patients with distant metastasis at initial diagnosis: therapeutic approaches and prognostic factors. *PLoS One* 2014;9(9):e108070.

7. Chen M, Jiang R, Guo L, et al. Locoregional radiotherapy in patients with distant metastases of nasopharyngeal carcinoma at diagnosis. *Chin J Cancer* 2013;32(11):604-613.

8. Lin SJ, Tham IWK, Pan JJ, et al. Combined high-dose radiation therapy and systemic chemotherapy improves survival in patients with newly diagnosed metastatic nasopharyngeal cancer. *Am J Clin Oncol* 2012;35(5):474-479.

9. Yeh SA, Tang Y, Lui CC, et al. Treatment outcomes of patients with AJCC stage IVC nasopharyngeal carcinoma: benefits of primary radiotherapy. *Jpn J Clin Oncol* 2006;36(3):132-136.
